# Different Evolutionary Trends of Galloanseres: Mitogenomics Analysis

**DOI:** 10.3390/ani14101437

**Published:** 2024-05-11

**Authors:** Shengyang Zhou, Xibao Wang, Lidong Wang, Xiaodong Gao, Tianshu Lyu, Tian Xia, Lupeng Shi, Yuehuan Dong, Xuesong Mei, Zhihao Zhang, Honghai Zhang

**Affiliations:** College of Life Sciences, Qufu Normal University, Jingxuan West Street No. 57, Qufu 273165, China; zhoushengyang94@163.com (S.Z.); wangxibao1995@163.com (X.W.); wanglidong955@163.com (L.W.); gao-xiaodong@163.com (X.G.); 17865717265@163.com (T.L.); qfxiatian1993@163.com (T.X.); shilupeng19961105@163.com (L.S.); dongyuehuan2019@163.com (Y.D.); meixsunique@126.com (X.M.); zhangzhihao1102@163.com (Z.Z.)

**Keywords:** Galloanseres, Galliformes, Anseriformes, mitochondria, mitogenome, phylogenetic, biological evolution, evolutionary trends, energy requirements

## Abstract

**Simple Summary:**

Galloanseres are one of the most widely distributed groups of birds, which occupy different ecological niches and exhibit different evolutionary trends. Mitochondria are widely used in phylogenetic analysis and ecological research due to unique genetic mechanisms and significant physiological functions. Adapting to different environments requires meeting varying energy demands, which is closely related to mitochondria. In this study, we constructed two complete mitogenomes of *Aythya baeri* and *Aythya marila*. The phylogenetic and divergence time analysis based on the mitochondrial PCGs were conducted to elucidate the evolutionary process of species within Galloanseres. Additionally, the analysis of selective pressures between the two sister clades, Galliformes and Anseriformes, revealed that different evolutionary directions have shaped distinct evolutionary patterns of mitochondrial genes.

**Abstract:**

The two existing clades of Galloanseres, orders Galliformes (landfowl) and Anseriformes (waterfowl), exhibit dramatically different evolutionary trends. Mitochondria serve as primary sites for energy production in organisms, and numerous studies have revealed their role in biological evolution and ecological adaptation. We assembled the complete mitogenome sequences of two species of the genus *Aythya* within Anseriformes: *Aythya baeri* and *Aythya marila*. A phylogenetic tree was constructed for 142 species within Galloanseres, and their divergence times were inferred. The divergence between Galliformes and Anseriformes occurred ~79.62 million years ago (Mya), followed by rapid evolution and diversification after the Middle Miocene (~13.82 Mya). The analysis of selective pressure indicated that the mitochondrial protein-coding genes (PCGs) of Galloanseres species have predominantly undergone purifying selection. The free-ratio model revealed that the evolutionary rates of *COX1* and *COX3* were lower than those of the other PCGs, whereas *ND2* and *ND6* had faster evolutionary rates. The CmC model also indicated that most PCGs in Anseriformes exhibited stronger selective constraints. Our study suggests that the distinct evolutionary trends and energy requirements of Galliformes and Anseriformes drive different evolutionary patterns in the mitogenome.

## 1. Introduction

The Galloanseres, an ancient clade among extant birds, which together with the Neoaves forms the clade Neognathae, clusters with Paleognathae to form the three major group of modern birds (Neornithes) [1]. This clade consists of the orders Anseriformes and Galliformes and includes 142 genera and 480 species of birds worldwide. Birds of Galloanseres are distributed worldwide and occupy important ecological niches in various ecosystems. They have demonstrated different evolutionary trends. Species within Anseriformes, which are predominantly aquatic, commonly inhabit wetlands, streams, and lakes [2]. They excel at swimming and possess the ability to undertake long-distance migration. Conversely, species within Galliformes are primarily terrestrial, mostly inhabiting terrestrial environments, such as forests and grasslands, with limited flying ability [3]. Birds in this clade are also closely related to humans, and many domestic poultry species have been domesticated from them [4,5].

Mitochondria are important functional organelles in eukaryotes that provide most of the adenosine triphosphate (ATP) required for cells by means of oxidative phosphorylation (OXPHOS) [6,7] and play a central role in apoptotic cell death by altering mitochondrial outer membrane permeabilization [8]. In addition, mitochondria are involved in processes, such as fatty acid oxidation, phospholipid synthesis, the generation and maintenance of reactive oxygen species, and signaling in innate immunity [9,10], coordinating various metabolic processes, which are achieved through establishing contacts with other organelles, shedding specialized vesicles, or producing and releasing of signaling metabolites [11]. Mitochondria can maintain their integrity and homeostasis through fusion and fission according to the requirements of the cell [12], thereby affecting the aforementioned processes. These are significant for organisms to adapt to various ecological niches [13,14,15,16]. Vertebrate mitochondria possess an independent genetic system consisting of 37 genes, which includes 2 ribosomal RNA (rRNA) genes, 13 protein-coding genes (PCGs), 22 transfer RNA (tRNA) genes, and 1 non-coding control region (D-loop). Mitochondrial genes are ideal materials for studying animal phylogeny because of their extreme conservation, maternal inheritance, and non-recombination during generation transmission [17,18]. For example, Lebedev et al. elucidated the interspecific relationships within the Allactaginae subfamily by constructing a phylogenetic tree using both nuclear and mitochondrial genes [19]. Additionally, because mitochondria can provide the power needed for biological functions, they may offer insights into how organisms adapt to different ecological niches. Some studies have shown that the mitochondrial genes of organisms living in different environments often have different evolutionary rates [20,21,22,23], which may be related to the survival pressure faced by organisms; that is, organisms require different energies to adapt to different environments. To date (April 2024), there have been no reports regarding the different evolutionary directions of species within Galloanseres based on mitogenomes. Considering the crucial role of mitochondria in the physiological activities and adaptation to different environments of organisms, we explored the phylogenetic relationships and adaptive evolution of Galloanseres, aiming to provide new insights into the evolutionary process and adaptive evolution of Galloanseres.

In the present study, we assembled and annotated two new mitogenomes of the genus *Aythya* (*Aythya baeri* and *Aythya marila*) based on high-quality nanopore sequencing data to provide new genetic resources for *Aythya*. To elucidate the evolutionary relationships within Galloanseres, we constructed a phylogenetic tree for 142 birds of Galloanseres based on their PCGs and inferred their divergence times. We also analyzed the selection pressure on the PCGs of species within Galloanseres to elucidate their roles in species adaptation to different ecological niches. Our study provides insights into the phylogenetic relationships among Galloanseres and their adaptations to different ecological niches.

## 2. Materials and Methods

### 2.1. Assembly and Annotation of the Mitogenome

We assembled two complete mitogenomes of *Aythya* using NOVOPlasty version 4.3.1 [24]. The raw data of genome sequencing used (*A. baeri*, SRR17568785; *A. marila*, SRR21672224; sequenced by our research group) were downloaded from the NCBI SRA database. The seed sequence used was the complete mitogenome of *Anas platyrhynchos* (NC_009684.1), and the K-mer and genome range were set to 33 and 12,000–20,000, respectively. We validated and revised the assembled sequences by comparing them with the seed sequence using BLAST and annotated the mitogenomes using MITOS2 [25] (http://mitos2.bioinf.uni-leipzig.de/index.py, accessed on 26 November 2022). The complete mitogenomes assembled were submitted to the NCBI GenBank database under accession numbers OP909754 (*A. marila*) and OP909755 (*A. baeri*). OGDRAW [26] of the MPI-MP CHLOROBOX website (https://chlorobox.mpimp-golm.mpg.de/geseq.html, accessed on 23 April 2024) was used to construct a gene map of these mitogenomes. The relative synonymous codon usage (RSCU) of mitochondrial PCGs was calculated using MEGA X [27]. The RSCU was plotted using the R software package version 4.1.3 (package, ggplot2). Nucleotide composition skew was calculated using the following formula: AT-skew = (A −  T)/(A + T) and GC-skew = (G − C)/(G + C).

### 2.2. Phylogenetic Analysis and Divergence Time Inference

In addition to the two mitogenomes of *Aythya*, we downloaded the published complete mitogenomes of all species within Galloanseres and the rock dove (*Columba livia*) from GenBank (July 2023). The reference or longest sequence of each species was used for phylogenetic analysis (Table S1). We used PhyloSuite version 1.2.3 [28] to extract the 13 PCGs (*COX1*, *COX2*, *COX3*, *ND1*, *ND2*, *ND3*, *ND4*, *ND4L*, *ND5*, *ND6*, *ATP6*, *ATP8*, and *CYTB*), MAFFT version 7.505 [29] to align them, and Gblocks version 0.91b [30] to extract conserved sites from the multiple sequence alignment results. The conserved sequences of all species were concatenated into a supermatrix to infer the phylogenetic relationships. Based on the Bayesian information criterion, ModelFinder version 2.2.0 [31] was used to identify the best model (GTR+F+I+G4) for MrBayes version 3.2.7a [32]. MrBayes was used to generate a phylogenetic tree with Markov chain Monte Carlo (MCMC) chains; the generation parameter was set to 2,000,000 and sampled every 1000 generations; and the burnin fraction was set to 25.0% for diagnostics. A rock dove was used as an outgroup.

The time of species divergence was inferred using the MCMCtree module in the PAML version 4.7 [33] and the first recorded time for the families Anatidae and Phasianidae (Anatidae, 40.4–46.2 million years ago [Mya]; Phasianidae, 41.3–47.8 Mya) on the mindat.org website (https://www.mindat.org, accessed on 6 June 2023), and the divergence times of Anseriformes and Galliformes (72.5–85.4 Mya) from TimeTree [34] were used as correction time.

### 2.3. Selection Pressure Analysis of Mitochondrial Genes

Synonymous and non-synonymous mutations of organism proteins occur under different selection pressures and are fixed at different rates [33]. Therefore, we used the ratio of non-synonymous mutations (*dN*) to synonymous mutations (*dS*) (*ω* = *dN/dS*) to reveal the intensity of natural selection on PCGs. The codeml module in PAML was used to analyze natural selection pressure. The free-ratio model (model = 1, NSsites = 0; null model, M0, model = 0, NSsites = 0) allows each branch to have an independent *ω* value, enabling the assessment of the natural selection pressure on each terminal branch. Some branches may lack synonymous or non-synonymous substitutions, which could lead to extreme *ω* estimates [35]; in such cases, we designated the *ω* value as “NA”. To assess the phylogenetic evolutionary rates of Anseriformes and Galliformes, we used Clade model C (CmC model, model = 3, NSsites = 2, ncatG = 3; null model, M2a_rel, model = 0, NSsites = 22), which could simultaneously detect selection pressures on multiple particular lineages.

## 3. Results

### 3.1. Mitogenome Structure and Annotation

**Figure 1:**
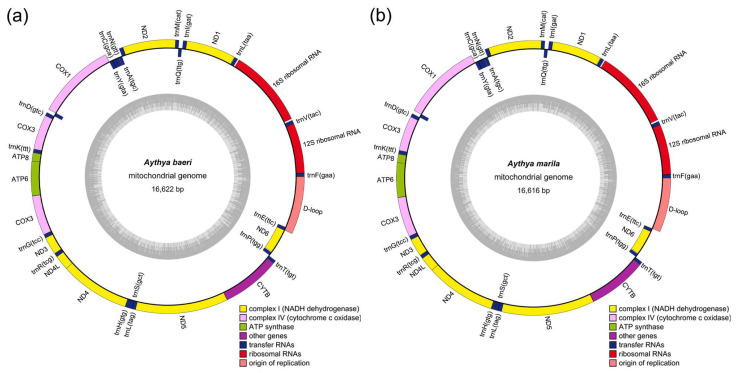
Mitogenome gene map of *A. baeri* (**a**) and *A. marila* (**b**). The genes outside the circle are transcribed clockwise, whereas the genes inside the circle are transcribed counterclockwise.

We assembled the complete mitogenomes of *A. baeri* and *A. marila*, which were 16,622 and 16,616 bp in size, respectively. The GC contents of the complete mitogenomes of *A. marila* and *A. baeri* were 48.3% and 48.1%, respectively, which are similar to that of other species within Anatidae and slightly lower than that of AT bases (Table S1). The AT skew values of *A. marila* and *A. baeri* were 0.139 and 0.143, respectively, whereas the GC skew values were −0.359 and −0.362, indicating a higher content of the A bases than the T bases and a higher content of the C bases than the G bases. Both complete mitogenomes contained 37 genes, including 13 PCGs, 22 tRNA genes, 2 rRNA genes, and 1 non-coding region (Figure 1a,b). The *ND6* gene and 8 tRNA genes (*trnQ*, *trnA*, *trnN*, *trnC*, *trnY*, *trnS*, *trnP*, *ND6*, and *trnE*) are located on the light chains, whereas the remaining 28 genes are located on the heavy strand (Table 1 and Table 2).

**Figure 2:**
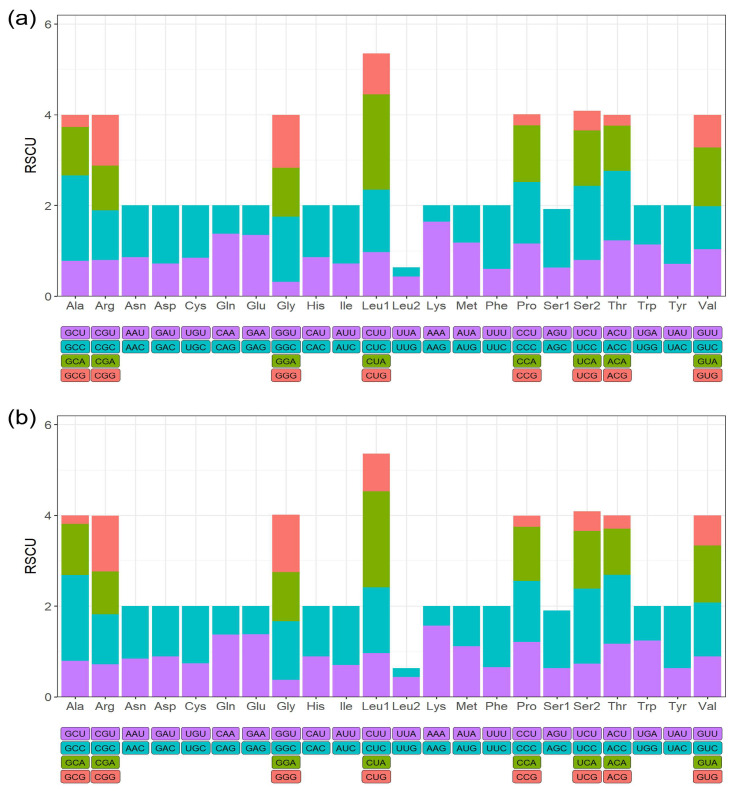
Relative synonymous codon usage (RSCU) of *A. baeri* (**a**) and *A. marila* (**b**). The X-axis shows the codon family types, and the Y-axis shows the proportion of each codon type to the respective codon family.

Across the mitogenome sequences, there were 34 bp in overlapping regions, and both species exhibited the largest overlap of 10 bp between *ATP8* and *ATP6* (Table 1 and Table 2). Both species shared the same start and stop codons in their PCGs (Table 1 and Table 2). Apart from the conventional start codon ATG, which was present in 10 PCGs, the non-standard codon GTG was found in *COX1*, *COX2*, and *ND5*. Four types of stop codons were identified: AGG, TAA, TAG, and T. Codon usage analysis revealed that these two species have strong preferences for eight codon families: Ala, Arg, Gly, Leu1, Pro, Ser2, Thr, and Val (Figure 2a,b).

### 3.2. Phylogenetic and Divergence Time Analysis

**Figure 3:**
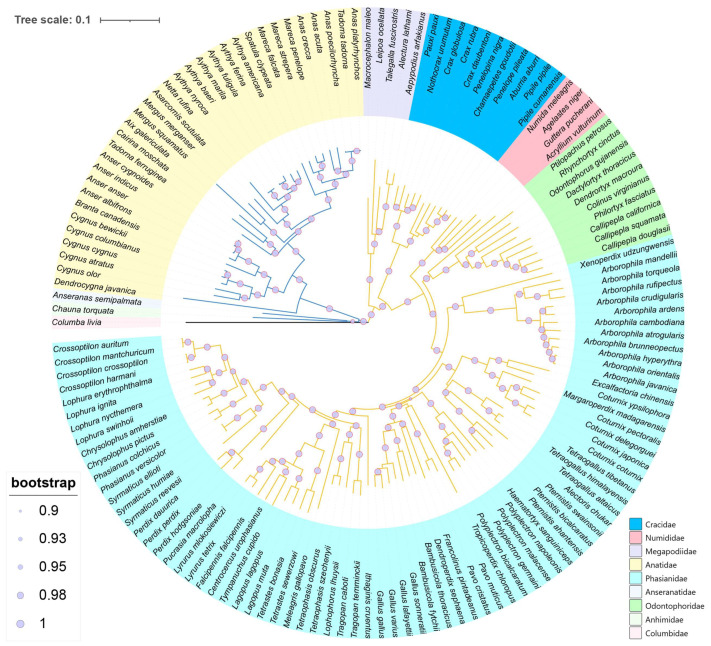
The Bayesian inference of phylogenetic trees for 143 species based on PCG matrix.

We present a Bayesian inference phylogenetic tree with high bootstrap support values and Bayesian posterior probabilities for 142 species of Galloanseres (Figure 3). The minimum and average effective sample size (ESS) were 1297.82 and 1384.62, respectively, indicating that the Bayesian inference of phylogeny was reliable. The phylogenetic relationships showed that Galloanseres are a monophyletic group divided into two evolutionary clades: Anseriformes and Galliformes. Species of Anseriformes and Galliformes are clustered together. Within Anseriformes, the closest relationship is observed between the family Anhimidae and Anseranatidae, which then cluster together with Anatidae. *A. baeri* and *A. marila* are most closely related to *Aythya nyroca* and *Aythya fuligula*, respectively. *Tadorna tadorna* clusters together with *Anas platyrhynchos* rather than grouping with *Tadoma ferruginea* from the same genus (*Tadorna*) because this species should be identified as the Linwu duck (*Anas platyrhynchos domestica*) [36]. Within Galliformes, the family Phasianidae is most closely related to the family Odontophoridae, followed by the clade leading to the families Numididae, Cracidae, and Megapodiidae. Our results exhibited a topology similar to those of previous studies [37,38].

**Figure 4:**
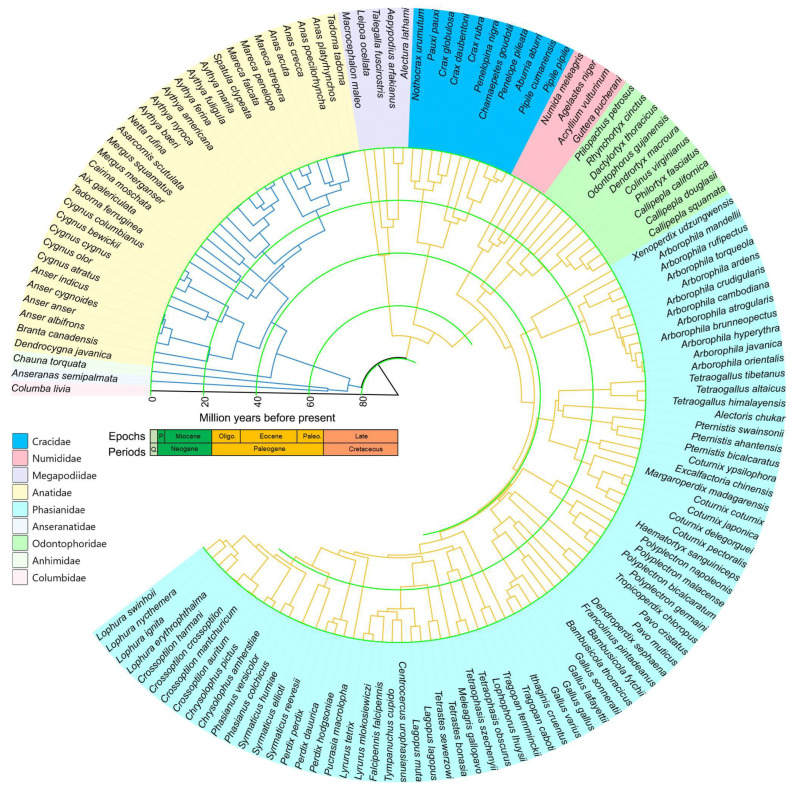
Divergence time estimation of 143 Species based on 13 mitochondrial PCGs. “Q.” is an abbreviation for Quaternary; “P.” is an abbreviation for Pliocene.

Based on fossil-calibrated species divergence times (Figure 4), Galliformes and Anseriformes diverged at approximately 79.62 Mya (95% CI: 73.45–85.23 Mya). The main clade of Anseriformes, family Anatidae, split around 74.54 Mya (95% CI: 68.89–80.06 Mya), whereas another clade split into Anseranatidae and Anhimidae around 67.07 Mya (95% CI: 61.19–73.02 Mya). The separation times of several major families within Galliformes are as follows: Megapodiidae around 71.33 Mya (95% CI: 66.18–76.62), Cracidae around 66.48 Mya (95% CI: 61.81–71.59), Numididae around 49.71 Mya (95% CI: 47.23–54.16), and the divergence of Phasianidae and Odontophoridae around 47.14 Mya (95% CI: 45.03–51.51). Simultaneously, it is noteworthy that Galloanseres continued to diversify rapidly after the Middle Miocene.

### 3.3. Selection Pressure Analysis

**Figure 5:**
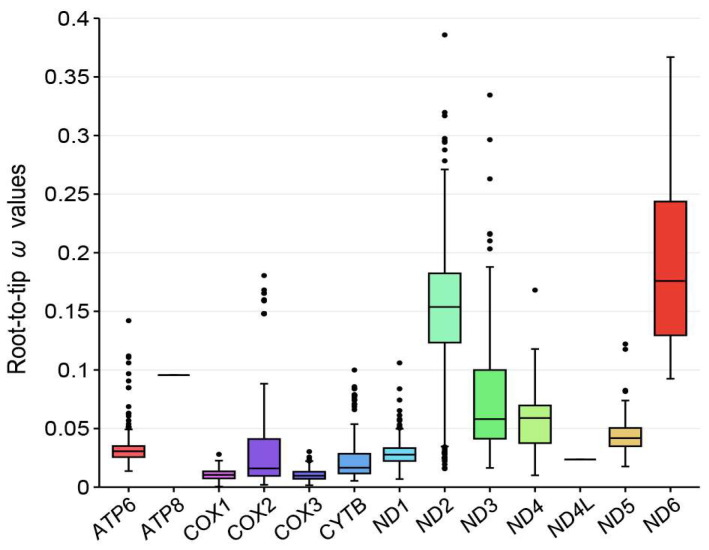
Box plot of 13 mitochondrial genes’ evolution rates (*ω*).

The results of the free-ratio model indicated that, with the exception of *ATP8* and *ND4L*, all other genes were better explained by the free-ratio model than by the M0 model (*p* < 0.05) (Table S2). *ATP8* and *ND4L* showed the same rate of evolution across all species, whereas the other genes exhibited different rates of evolution. Most species’ mitochondrial PCGs have undergone purifying selection (*ω* < 1), except for *Arborophila orientalis*, where a signal of positive selection was detected in *ND4* (*ω* = 1.172) (Table S2). We used the root-to-tip *ω* to assess the selection pressure of different mitochondrial PCGs. Overall, larger *ω* values were observed in the Galloanseres species for *ND2* and *ND6*, whereas smaller *ω* values were evident for *COX1* and *COX3* (Figure 5 and Table S3). These indicated that the *ND2* and *ND6* likely evolved more quickly, whereas the *COX1* and *COX3* were more conservative.

We used the CmC model to examine the evolutionary rates of the two clades, and the results indicated differences in the evolutionary rates between lineages occupying the two distinct ecological niches (Table 3). Likelihood ratio tests showed no significant (*p* > 0.05) differences in *ATP8*, *COX2*, *ND4L*, or *ND6* between the two models, indicating that the evolutionary rates of the two clades were the same, which supports the outcome of the null model. Other genes showed that the M2a_rel model was superior to the CmC model. The *ω* values of all genes were <1, indicating that these genes were under purifying selection pressure in both Galliformes and Anseriformes. For the other genes, except for *ATP6*, the evolutionary rate was lower in Anseriformes than in Galliformes.

## 4. Discussion

The adaptive evolution of organisms is a complex process that involves multiple processes, such as genetics, transcription, metabolism, and physiological activities. Maternally inherited, possessing independent genetic and proteosynthetic apparatus, providing most of the energy required for life activities, and participating in a multitude of life processes, these making mitochondria ideal materials for studying phylogeny and adaptive evolution. In this study, we present a comparative analysis of the mitogenomes in Galloanseres to investigate their roles in the divergent evolutionary trajectories of the two sister clades and the evolutionary process of species within Galloanseres.

We assembled the complete mitogenomes of *A. baeri* and *A. marila* using NOVOPlasty, based on high-quality, high-throughput sequencing data. The mitogenomes of these two diving ducks exhibited typical circular structures with lengths of 16,622 and 16,616 bp, respectively, and contained 13 PCGs, 22 tRNA genes, 2 rRNA genes, and 1 non-coding region. The gene structures and lengths were similar to those of the mitogenomes of other species within *Aythya* reported in previous studies (Table 1, Table 2 and Table S1) [39,40,41,42,43]. Additionally, differences in nucleotide composition and codon usage were extremely similar, indicating a highly conserved mitogenome structure among species within *Aythya*. Although mitochondria are widely present in eukaryotic cells, they possess independent DNA and translation systems, with translation types similar to those of bacteria [44,45]. Two start codons were observed within these two mitogenomes: GTG and the standard codon ATG, which are also relatively common start codons in vertebrates [46,47,48,49]. There are multiple types of stop codons, including AGG, TAA, TAG, and an incomplete codon T. AGG serves as a codon for encoding arginine, but in mitochondria, owing to the absence of cognate tRNAs, this codon is reassigned to function as a stop codon [50] and terminates translation by interacting with mitochondrial release factor 1 [51,52]. The incomplete T stop codon could be completed by means of posttranscriptional polyadenylation [20].

Through phylogenetic analysis, we constructed a robust phylogenetic tree for Galloanseres, with each node being well supported (bootstrap > 0.9). This phylogenetic tree covered all families and more than half (75 genera) of the 142 genera in Galloanseres, providing a comprehensive explanation of the phylogenetic relationships within the subclass. According to the species divergence time estimation, the two clades of Galloanseres diverged around 79.62 Mya, and it was not until the past few million years that the major lineages of Galloanseres gradually formed. Since the Middle Miocene (~13.82 Mya), species of Galloanseres have undergone rapid evolution and diversification. This could be the result of climate change, causing the fragmentation and vicariance of biological communities [53]. The Miocene climatic optimum promoted population expansion, and the cooling episode during the Middle Miocene led to a wave of retraction and fragmentation of biological communities [54], increasing the opportunity for the formation of new isolated populations [55].

Mitochondrial OXPHOS is at the core of cellular energy metabolism, which is crucial for energy generation in eukaryotic organisms. The analysis of selective pressures on the 13 mitochondrial PCGs revealed the roles and contributions of these genes in adaptation to different ecological niches. Both the free-ratio model and CmC model analysis showed evidence of purifying selection acting on these genes (except for *ND4* in *A. orientalis*), indicating that mutations in mitochondrial genes are mostly synonymous. Studies in other organisms have suggested that mitochondrial genes primarily undergo purifying selection [21,23,56,57]. Non-synonymous mutations involve the replacement of amino acid residues, leading to variants with uncertain significance [58], which can result in OXPHOS dysfunction and related diseases [59], whereas synonymous mutations can maintain the normal structure and function of proteins. Mitochondrial genes also exhibit different evolutionary patterns across species. Cytochrome c oxidase is a key rate-limiting enzyme in the electron transport chain that drives OXPHOS and serves as a regulatory center for oxidative phosphorylation [60]. In addition, this enzyme is subjected to feedback inhibition by ATP to prevent the formation of reactive oxygen species. Two genes encoding cytochrome c oxidase subunits (*COX1* and *COX3*) show strong purifying selection, which may be related to their essential functions. NADH dehydrogenase (complex I) is the most complicated enzyme complex of the mitochondrial respiratory chain, encoded by seven mitochondrial genes [61]. *ND2* and *ND6* may have experienced more relaxed selective constraints, which allowed them to accumulate more mutations, but these may not affect the normal function of NADH dehydrogenase. In general, the mitochondrial genes of Galliformes have exhibited a faster rate of evolution. Our evaluation of the evolutionary rates of both the Galliformes and Anseriformes clades largely supported this result. Different evolutionary directions impose selective constraints on the mitogenome, leading to different rates of evolution. Birds of Anseriformes, which require swimming and long-distance flight, have stronger locomotor abilities than birds of Galliformes. Stronger purifying selection may help birds of Anseriformes maintain an efficient energy metabolism, whereas birds of Galliformes relax their energy requirements, allowing them to experience more relaxed selective pressures [62].

## 5. Conclusions

We assembled two new complete mitogenome sequences of species within *Aythya* based on high-quality sequencing data, estimated a robust phylogenetic relationship tree within the Galloanseres, and inferred divergence times among species based on fossil calibrations. We found that species within Galloanseres underwent rapid evolution and diversification after the Middle Miocene. The analysis of the selection pressure on mitochondrial PCGs indicated that purifying selection was the predominant trend in Galloanseres, which may be crucial for maintaining normal OXPHOS. The evolutionary rates of *COX1* and *COX3* were slower than those of other genes, likely because of their important functions in the electron transport chain. The evolutionary rate of mitochondrial PCGs in Anseriformes was generally lower than that in Galliformes, indicating that different evolutionary directions with varying energy demands influenced the mitochondrial evolution rate. In summary, our study provides new genetic resources for *A. baeri* and *A. marila*, revealing the phylogenetic relationships and evolutionary processes of species within Galloanseres, and providing molecular evidence for the evolution of Galloanseres.

## Figures and Tables

**Table 1:** Mitogenome characteristics of *A. baeri*.

Gene	Nucleotide Positions	Size (bp)	Stand	Intergenic Nucleotide	Start	Stop
*trnF*	1–70	70	+			
*12s rRNA*	70–1053	984	+	−1		
*trnV*	1054–1124	71	+	0		
*16s rRNA*	1155–2704	1550	+	30		
*trnL*	2729–2802	74	+	24		
*ND1*	2807–3784	978	+	4	ATG	AGG
*trnI*	3783–3854	72	+	−2		
*trnQ*	3863–3933	71	−	8		
*trnM*	3933–4001	69	+	−1		
*ND2*	4002–5040	1039	+	0	ATG	T
*trnW*	5041–5116	76	+	0		
*trnA*	5120–5188	69	−	3		
*trnN*	5191–5263	73	−	2		
*trnC*	5264–5329	66	−	0		
*trnY*	5329–5399	71	−	−1		
*COX1*	5401–6951	1551	+	1	GTG	AGG
*trnS*	6943–7015	73	−	−9		
*trnD*	7018–7086	69	+	2		
*COX2*	7088–7774	687	+	1	GTG	TAA
*trnK*	7776–7843	68	+	1		
*ATP8*	7845–8012	168	+	1	ATG	TAA
*ATP6*	8003–8686	684	+	−10	ATG	TAA
*COX3*	8686–9469	784	+	−1	ATG	T
*trnG*	9470–9538	69	+	0		
*ND3*	9539–9890	352	+	0	ATG	TAA
*trnR*	9892–9962	71	+	1		
*ND4L*	9963–10,259	297	+	0	ATG	TAA
*ND4*	10,253–11,630	1378	+	−7	ATG	T
*trnH*	11,631–11,699	69	+	0		
*trnS*	11,700–11,765	66	+	0		
*trnL*	11,765–11,835	71	+	−1		
*ND5*	11,836–13,659	1824	+	0	GTG	TAA
*CYTB*	13,659–14,801	1143	+	−1	ATG	TAA
*trnT*	14,804–14,872	69	+	2		
*trnP*	14,883–14,952	70	−	10		
*ND6*	14,963–15,484	522	−	10	ATG	TAG
*trnE*	15,485–15,552	68	−	0		

**Table 2:** Mitogenome characteristics of *A. marila*.

Gene	Nucleotide Positions	Size (bp)	Stand	Intergenic Nucleotide	Start	Stop
*trnF*	1–70	70	+			
*12s rRNA*	70–1054	985	+	−1		
*trnV*	1055–1125	71	+	0		
*16s rRNA*	1156–2705	1550	+	30		
*trnL*	2729–2802	74	+	23		
*ND1*	2807–3784	978	+	4	ATG	AGG
*trnI*	3783–3854	72	+	−2		
*trnQ*	3863–3933	71	−	8		
*trnM*	3933–4001	69	+	−1		
*ND2*	4002–5040	1039	+	0	ATG	T
*trnW*	5041–5116	76	+	0		
*trnA*	5120–5188	69	−	3		
*trnN*	5191–5263	73	−	2		
*trnC*	5264–5329	66	−	0		
*trnY*	5329–5399	71	−	−1		
*COX1*	5401–6951	1551	+	1	GTG	AGG
*trnS*	6943–7015	73	−	−9		
*trnD*	7018–7086	69	+	2		
*COX2*	7088–7774	687	+	1	GTG	TAA
*trnK*	7776–7843	68	+	1		
*ATP8*	7845–8012	168	+	1	ATG	TAA
*ATP6*	8003–8686	684	+	−10	ATG	TAA
*COX3*	8686–9469	784	+	−1	ATG	T
*trnG*	9470–9538	69	+	0		
*ND3*	9539–9890	352	+	0	ATG	TAA
*trnR*	9892–9961	70	+	1		
*ND4L*	9962–10,258	297	+	0	ATG	TAA
*ND4*	10,252–11,629	1378	+	−7	ATG	T
*trnH*	11,630–11,698	69	+	0		
*trnS*	11,699–11,764	66	+	0		
*trnL*	11,764–11,834	71	+	−1		
*ND5*	11,835–13,658	1824	+	0	GTG	TAA
*CYTB*	13,658–14,800	1143	+	−1	ATG	TAA
*trnT*	14,803–14,871	69	+	2		
*trnP*	14,882–14,951	70	−	10		
*ND6*	14,962–15,483	522	−	10	ATG	TAG
*trnE*	15,484–15,551	68	−	0		

**Table 3:** Positive selection test of mitochondrial PCGs in different clades using the CmC model.

Gene	Model Compared	|2ΔlnL|	*p*-Value	M2a_rel	CmC	
				*ω*	*ω*_ANS_	*ω*_GAL_
*ATP6*	CmC vs. M2a_rel	15.9197	3.492 × 10^−4^ **	0.1565	0.1679	0.1582
*ATP8*		4.2318	0.1205	0.2575	0.1702	0.2763
*COX1*		13.7733	0.0010 **	0.1073	0.1034	0.1099
*COX2*		1.5259	0.4663	0.1133	0.1290	0.1109
*COX3*		8.0060	0.0183 *	0.0633	0.0613	0.0660
*CYTB*		12.8937	0.0016 **	0.0886	0.0832	0.0918
*ND1*		13.7817	0.0010 **	0.1661	0.1557	0.1697
*ND2*		60.4582	7.438 × 10^−14^ **	0.1537	0.1038	0.1696
*ND3*		12.5340	0.0019 **	0.1720	0.1232	0.1907
*ND4*		26.8034	1.513 × 10^−6^ **	0.1313	0.1074	0.1402
*ND4L*		3.3608	0.1863	0.1003	0.1267	0.0936
*ND5*		16.7277	2.33 × 10^−4^ **	0.1346	0.1075	0.1443
*ND6*		8.9346	0.0115 *	0.1288	0.1004	0.1388

Note: *, significant level (* *p* < 0.05, ** *p* < 0.01). ANS: the clade of Anseriformes; GAL, the clade of Galliformes.

## Data Availability

All the mitochondria genomes sequences used in this study were accessed through the GenBank database using the accession numbers in Table 1.

## Supplementary Materials

The following supporting information can be downloaded at: https://www.mdpi.com/article/10.3390/ani14101437/s1, Table S1: Information of all the mitochondrial genomes used; Table S2: Positive selection test of mitochondrial PCGs in different branches using the free-ratio model; Table S3: The root-to-tip ω of mitochondrial PCGs.

## References

[1] Jarvis E.D., Mirarab S., Aberer A.J., Li B., Houde P., Li C., Ho S.Y., Faircloth B.C., Nabholz B., Howard J.T. (2014). Whole-genome analyses resolve early branches in the tree of life of modern birds. Science.

[2] De Mendoza R.S., Gomez R.O. (2022). Ecomorphology of the tarsometatarsus of waterfowl (Anseriformes) based on geometric morphometrics and its application to fossils. Anat. Rec..

[3] Hosner P.A., Tobias J.A., Braun E.L., Kimball R.T. (2017). How do seemingly non-vagile clades accomplish trans-marine dispersal? Trait and dispersal evolution in the landfowl (Aves: Galliformes). Proc. Biol. Sci..

[4] Sarkar I., Rathore S.S., Singh R.P. (2021). An interplay between compositional constraint and natural selection dictates the codon usage pattern among select Galliformes. Bio Syst..

[5] Cordero G.A., Werneburg I. (2022). Domestication and the comparative embryology of birds. J. Exp. Zool. B Mol. Dev. Evol..

[6] Vercellino I., Sazanov L.A. (2022). The assembly, regulation and function of the mitochondrial respiratory chain. Nat. Rev. Mol. Cell Biol..

[7] Sousa J.S., D’Imprima E., Vonck J. (2018). Mitochondrial Respiratory Chain Complexes. Subcell. Biochem..

[8] Bock F.J., Tait S.W.G. (2020). Mitochondria as multifaceted regulators of cell death. Nat. Rev. Mol. Cell Biol..

[9] Ng M.Y.W., Wai T., Simonsen A. (2021). Quality control of the mitochondrion. Dev. Cell.

[10] Shadel G.S., Horvath T.L. (2015). Mitochondrial ROS signaling in organismal homeostasis. Cell.

[11] Collier J.J., Olahova M., McWilliams T.G., Taylor R.W. (2023). Mitochondrial signalling and homeostasis: From cell biology to neurological disease. Trends Neurosci..

[12] Adebayo M., Singh S., Singh A.P., Dasgupta S. (2021). Mitochondrial fusion and fission: The fine-tune balance for cellular homeostasis. FASEB J..

[13] Mallet R.T., Burtscher J., Pialoux V., Pasha Q., Ahmad Y., Millet G.P., Burtscher M. (2023). Molecular Mechanisms of High-Altitude Acclimatization. Int. J. Mol. Sci..

[14] Hood W.R., Austad S.N., Bize P., Jimenez A.G., Montooth K.L., Schulte P.M., Scott G.R., Sokolova I., Treberg J.R., Salin K. (2018). The Mitochondrial Contribution to Animal Performance, Adaptation, and Life-History Variation. Integr. Comp. Biol..

[15] O’Brien K.M. (2011). Mitochondrial biogenesis in cold-bodied fishes. J. Exp. Biol..

[16] Bennett C.F., Latorre-Muro P., Puigserver P. (2022). Mechanisms of mitochondrial respiratory adaptation. Nat. Rev. Mol. Cell Biol..

[17] Francoso E., Zuntini A.R., Ricardo P.C., Santos P.K.F., de Souza Araujo N., Silva J.P.N., Goncalves L.T., Brito R., Gloag R., Taylor B.A. (2023). Rapid evolution, rearrangements and whole mitogenome duplication in the Australian stingless bees Tetragonula (Hymenoptera: Apidae): A steppingstone towards understanding mitochondrial function and evolution. Int. J. Biol. Macromol..

[18] Sasaki T., Sato M. (2021). Degradation of paternal mitochondria via mitophagy. Biochim. Biophys. Acta Gen. Subj..

[19] Lebedev V.S., Shenbrot G.I., Krystufek B., Mahmoudi A., Melnikova M.N., Solovyeva E.N., Lisenkova A.A., Undrakhbayar E., Rogovin K.A., Surov A.V. (2022). Phylogenetic relations and range history of jerboas of the Allactaginae subfamily (Dipodidae, Rodentia). Sci. Rep..

[20] Shang Y., Wang X., Liu G., Wu X., Wei Q., Sun G., Mei X., Dong Y., Sha W., Zhang H. (2022). Adaptability and Evolution of Gobiidae: A Genetic Exploration. Animals.

[21] Wang X., Zhou S., Wu X., Wei Q., Shang Y., Sun G., Mei X., Dong Y., Sha W., Zhang H. (2021). High-altitude adaptation in vertebrates as revealed by mitochondrial genome analyses. Ecol. Evol..

[22] Zhao B., Gao S., Zhao M., Lv H., Song J., Wang H., Zeng Q., Liu J. (2022). Mitochondrial genomic analyses provide new insights into the “missing” atp8 and adaptive evolution of Mytilidae. BMC Genom..

[23] Wang X., Shang Y., Wu X., Wei Q., Zhou S., Sun G., Mei X., Dong Y., Sha W., Zhang H. (2023). Divergent evolution of mitogenomics in Cetartiodactyla niche adaptation. Org. Divers. Evol..

[24] Dierckxsens N., Mardulyn P., Smits G. (2017). NOVOPlasty: De novo assembly of organelle genomes from whole genome data. Nucleic Acids Res..

[25] Donath A., Juhling F., Al-Arab M., Bernhart S.H., Reinhardt F., Stadler P.F., Middendorf M., Bernt M. (2019). Improved annotation of protein-coding genes boundaries in metazoan mitochondrial genomes. Nucleic Acids Res..

[26] Greiner S., Lehwark P., Bock R. (2019). OrganellarGenomeDRAW (OGDRAW) version 1.3.1: Expanded toolkit for the graphical visualization of organellar genomes. Nucleic Acids Res..

[27] Kumar S., Stecher G., Li M., Knyaz C., Tamura K. (2018). MEGA X: Molecular Evolutionary Genetics Analysis across Computing Platforms. Mol. Biol. Evol..

[28] Zhang D., Gao F., Jakovlic I., Zou H., Zhang J., Li W.X., Wang G.T. (2020). PhyloSuite: An integrated and scalable desktop platform for streamlined molecular sequence data management and evolutionary phylogenetics studies. Mol. Ecol. Resour..

[29] Katoh K., Standley D.M. (2013). MAFFT multiple sequence alignment software version 7: Improvements in performance and usability. Mol. Biol. Evol..

[30] Talavera G., Castresana J. (2007). Improvement of phylogenies after removing divergent and ambiguously aligned blocks from protein sequence alignments. Syst. Biol..

[31] Kalyaanamoorthy S., Minh B.Q., Wong T.K.F., von Haeseler A., Jermiin L.S. (2017). ModelFinder: Fast model selection for accurate phylogenetic estimates. Nat. Methods.

[32] Ronquist F., Teslenko M., van der Mark P., Ayres D.L., Darling A., Hohna S., Larget B., Liu L., Suchard M.A., Huelsenbeck J.P. (2012). MrBayes 3.2: Efficient Bayesian phylogenetic inference and model choice across a large model space. Syst. Biol..

[33] Yang Z. (2007). PAML 4: Phylogenetic analysis by maximum likelihood. Mol. Biol. Evol..

[34] Kumar S., Suleski M., Craig J.M., Kasprowicz A.E., Sanderford M., Li M., Stecher G., Hedges S.B. (2022). TimeTree 5: An Expanded Resource for Species Divergence Times. Mol. Biol. Evol..

[35] Álvarez-Carretero S., Kapli P., Yang Z. (2023). Beginner’s Guide on the Use of PAML to Detect Positive Selection. Mol. Biol. Evol..

[36] Lin Q., Jiang G.T., Yun L., Li G.J., Dai Q.Z., Zhang S.R., Hou D.X., He X. (2016). The complete mitochondrial genome of the Linwu duck. Mitochondrial DNA A DNA Mapp. Seq. Anal..

[37] Sun Z., Pan T., Hu C., Sun L., Ding H., Wang H., Zhang C., Jin H., Chang Q., Kan X. (2017). Rapid and recent diversification patterns in Anseriformes birds: Inferred from molecular phylogeny and diversification analyses. PLoS ONE.

[38] Kimball R.T., Hosner P.A., Braun E.L. (2021). A phylogenomic supermatrix of Galliformes (Landfowl) reveals biased branch lengths. Mol. Phylogenet. Evol..

[39] Ding L., Zhang C., Chang Q., Yan L., Zhang B. (2016). The complete mitochondrial genome of *Aythya fuligula* (Anatidae: Aythya). Mitochondrial DNA A DNA Mapp. Seq. Anal..

[40] Zhou W., Zhang C., Chang Q., Yan L., Pan T., Zhang B. (2016). The complete mitochondrial genome of *Aythya ferina* (Anatidae: Aythya). Mitochondrial DNA A DNA Mapp. Seq. Anal..

[41] Zhai H., Li Z., Mi S., Meng D., Yu H., Teng L., Liu Z. (2021). The complete mitochondrial genome of the Ferruginous Duck (*Aythya nyroca*) from Ningxia, China. Mitochondrial DNA B Resour..

[42] Zhai H., Meng D., Li Z., Si Y., Yu H., Teng L., Liu Z. (2022). Complete mitochondrial genome of the common Pochard (*Aythya ferina*) from Ningxia Hui autonomous region, China. Mitochondrial DNA B Resour..

[43] Zhang L., Xia T., Gao X., Yang X., Sun G., Zhao C., Liu G., Zhang H. (2023). Characterization and Phylogenetic Analysis of the Complete Mitochondrial Genome of *Aythya marila*. Genes.

[44] Lind C., Sund J., Åqvist J. (2013). Codon-reading specificities of mitochondrial release factors and translation termination at non-standard stop codons. Nat. Commun..

[45] Huot J.L., Enkler L., Megel C., Karim L., Laporte D., Becker H.D., Duchene A.M., Sissler M., Marechal-Drouard L. (2014). Idiosyncrasies in decoding mitochondrial genomes. Biochimie.

[46] Ai W., Peng X., Huang X., Xiang D., Chen X. (2015). Complete mitochodrial genome of *Spinibarbus caldwelli* (Cypriniformes, Cyprinidae). Mitochondrial DNA.

[47] Jiang J.J., Xia E.H., Gao C.W., Gao L.Z. (2016). The complete mitochondrial genome of western painted turtle, *Chrysemys picta bellii* (Chrysemys, Emydidae). Mitochondrial DNA A DNA Mapp. Seq. Anal..

[48] Wang L.Y., Chai Y.L., Ma H.M. (2016). The complete sequence of the mitochondrial genome of Duroc pig (*Sus Scrofa*). Mitochondrial DNA A DNA Mapp. Seq. Anal..

[49] Zhao C., Zhang H., Zhang J., Chen L., Sha W., Yang X., Liu G. (2016). The complete mitochondrial genome sequence of the Tibetan wolf (*Canis lupus laniger*). Mitochondrial DNA A DNA Mapp. Seq. Anal..

[50] Anderson S., Bankier A.T., Barrell B.G., de Bruijn M.H., Coulson A.R., Drouin J., Eperon I.C., Nierlich D.P., Roe B.A., Sanger F. (1981). Sequence and organization of the human mitochondrial genome. Nature.

[51] Krüger A., Remes C., Shiriaev D.I., Liu Y., Spåhr H., Wibom R., Atanassov I., Nguyen M.D., Cooperman B.S., Rorbach J. (2023). Human mitochondria require mtRF1 for translation termination at non-canonical stop codons. Nat. Commun..

[52] Saurer M., Leibundgut M., Nadimpalli H.P., Scaiola A., Schönhut T., Lee R.G., Siira S.J., Rackham O., Dreos R., Lenarčič T. (2023). Molecular basis of translation termination at noncanonical stop codons in human mitochondria. Science.

[53] Claramunt S., Cracraft J. (2015). A new time tree reveals Earth history’s imprint on the evolution of modern birds. Sci. Adv..

[54] Methner K., Campani M., Fiebig J., Loffler N., Kempf O., Mulch A. (2020). Middle Miocene long-term continental temperature change in and out of pace with marine climate records. Sci. Rep..

[55] Vrba E.S. (1993). Turnover-pulses, the Red Queen, and related topics. Am. J. Sci..

[56] Wei Q., Wang X., Dong Y., Shang Y., Sun G., Wu X., Zhao C., Sha W., Yang G., Zhang H. (2023). Analysis of the Complete Mitochondrial Genome of *Pteronura brasiliensis* and *Lontra canadensis*. Animals.

[57] Wei Q., Zhang H., Wu X., Sha W. (2020). The selective constraints of ecological specialization in mustelidae on mitochondrial genomes. Mammal Res..

[58] Marsh J.A., Teichmann S.A. (2023). Predicting pathogenic protein variants. Science.

[59] Yan C., Duanmu X., Zeng L., Liu B., Song Z. (2019). Mitochondrial DNA: Distribution, Mutations, and Elimination. Cells.

[60] Kadenbach B. (2021). Complex IV—The regulatory center of mitochondrial oxidative phosphorylation. Mitochondrion.

[61] Ohnishi T., Ohnishi S.T., Salerno J.C. (2018). Five decades of research on mitochondrial NADH-quinone oxidoreductase (complex I). Biol. Chem..

[62] Shen Y.Y., Shi P., Sun Y.B., Zhang Y.P. (2009). Relaxation of selective constraints on avian mitochondrial DNA following the degeneration of flight ability. Genome Res..

